# Identification and in silico characterization of p.G380R substitution in FGFR3, associated with achondroplasia in a non-consanguineous Pakistani family

**DOI:** 10.1186/s13000-017-0642-3

**Published:** 2017-07-05

**Authors:** Muhammad Ajmal, Asif Mir, Muhammad Shoaib, Salman Akbar Malik, Muhammad Nasir

**Affiliations:** 1 Institute of Biomedical and Genetic Engineering, 24-Mauve area, G-9/1, Islamabad, 44000 Pakistan; 2 Department of Biotechnology, International Islamic University, Islamabad, Pakistan; 3 KRL General Hospital, Orthopedic Department, 24-Mauve area, G-9/1, Islamabad, 44000 Pakistan; 4 Department of Biochemistry, Quaid-i-Azam University, Islamabad, 44000 Pakistan

**Keywords:** Achondroplasia, FGFR3, Transmembrane domain, Mutation, Pakistan

## Abstract

**Background:**

The dimerization efficiency of FGFR3 transmembrane domain plays a critical role in the formation of a normal skeleton through the negative regulation of bone development. Recently, gain-of-function mutations in the transmembrane domain of *FGFR3* has been described associated with an aberrant negative regulation, leading to the development of achondroplasia-group disorders, including achondroplasia (ACH), hypochondroplasia (HCH) and thanatophoric dysplasia (TD). Here, we describe a non-consanguineous Pakistani family with achondroplasia to explain hereditary basis of the disease.

**Methods:**

PCR-based linkage analysis using microsatellite markers was employed to localize the disease gene. Gene specific intronic primers were used to amplify the genomic DNA from all affected as well as phenotypically healthy individuals. Amplified PCR products were then subjected to Sanger sequencing and RFLP analysis to identify a potentially pathogenic mutation. The impact of identified mutation on FGFR3 protein’s structure and stability was highlighted through different bioinformatics tools.

**Results:**

Genetic screening of the family revealed a previously reported heterozygous c.1138 G > A (p.G380R) mutation in the coding exon 8 of *FGFR3* gene. Identified genetic variation was confirmed in all affected individuals while healthy individuals and controls were found genotypically normal. The results were further validated by RFLP analysis as c.1138 G > A substitution generates a unique recognition site for *SfcI* endonuclease. Following *SfcI* digestion, the electrophoretic pattern of three bands/DNA fragments for each patient is indicative of heterozygous status of the disease allele. In silico studies of the mutant FGFR3 protein predicted to adversely affect the stability of FGFR3 protein.

**Conclusions:**

Mutation in the transmembrane domain may adversely affect the dimerization efficiency and overall stability of the FGFR3, leading to a constitutively active protein. As a result, an uncontrolled intracellular signaling or negative bone growth regulation leads to achondroplasia. Our findings support the fact that p.G380R is a common mutation among diverse population of the world and like other countries, can be used as a molecular diagnosis marker for achondroplasia in Pakistan.

**Electronic supplementary material:**

The online version of this article (doi:10.1186/s13000-017-0642-3) contains supplementary material, which is available to authorized users.

## Background

Achondroplasia (ACH) is inherited as an autosomal dominant trait of skeletal dysplasia with an estimated incidence ranging from 1:10,000 to 1: 70,000 live births [1, 2]. It is known to be the most common cause of dwarfism characterized by short stature that particularly affects the appendicular skeleton (upper and lower limbs) and to less extent, axial skeleton (skull, vertebrae, ribs) of the body [3–5].

Clinically, ACH patients represent typical features including shortened arms and legs (especially the upper arm and thigh), bowed lower legs, disproportionately large head-to-body size, frontal bossing and midface retrusion or hypoplasia [5–7]. During infancy, hypotonia is the most prominent characteristic caused by delayed and abnormal development of motor milestone [8]. Despite all these clinical manifestations, individuals with ACH have normal life span and intelligence factor [9].

Over the last two decades, dominant gain-of-function mutations of the specific site in fibroblast growth factor receptor 3 (FGFR3) have been shown implicated in human skeletal dysplasias, including achondroplasia (ACH), hypochondroplasia (HCH), thanatophoric dysplasia (TD) and severe achondroplasia, with developmental delay and acanthosis nigricans (SADDAN) [4, 10]. The FGFR3 is one of the four members of fibroblast growth factor receptor (FGFR) family, but differs from other FGFRs in its affinity for ligands and tissue distribution as it is mainly expressed in cartilage and brain [11, 12].

A typical FGFR contains an extracellular ligand-binding domain, a transmembrane region and an intracellular divided tyrosine kinase domain. Fibroblast growth factor (FGF) binds to an extracellular ligand-binding domain to initiate FGF/FGFR signaling that induces the expression of cell cycle suppression genes to negatively regulate bone development. However, mutations in the *FGFR3* gene lead to a constitutively active FGFR3 protein. As a result, a cascade of uncontrollable signal transduction allows an aberrant expression of the suppression genes, hence development of short stature pathology [10].

Almost 98% of the ACH cases are caused by variation at nucleotide position 1138, with 97% involving a c.1138 G > A mutation and 1% involving a c.1138 G > C mutation [13, 14]. Both mutations substitute glycine with arginine (p.G380R) in the transmembrane domain of FGFR3 protein that leads to gain-of-function [4, 15]. Mostly these mutations are de novo (sporadic) as more than 80% of ACH cases are born to their average-statured parents [16]. Advanced paternal age is one of the major reasons that significantly contribute to de-novo mutations in the germ cells because of large number of cell divisions during spermatogenesis [17]. Moreover, the presence of guanine at nucleotide position 1138, which is a part of CpG dinucleotide island and the most mutable site in the human genome, can also explain the high incidence of spontaneous mutations in *FGFR3* [18]. Other less frequent mutations are also identified in *FGFR3* but are mainly associated with hypochondroplasia and thanatophoric dysplasia type I and II [19]. Therefore, in comparison to other genetic diseases, ACH is a genetically and phenotypically homogenous disorder where very few rather than hundreds of mutations are responsible [20, 21]. In this study a non-consanguineous Pakistani family involving two affected generations, was clinically and genetically characterized for skeletal dysplasia. Genetic analysis revealed a heterozygous dominant mutation in *FGFR3* affecting the protein stability and dimerization efficiency, leading to ACH in a Pakistani family.

## Methods

### Subjects

A non-consanguineous Pakistan family with a history of ACH in two consecutive generations was identified from secluded area of KPK, Pakistan. Affected (*n* = 3) as well as phenotypically healthy individuals (*n* = 3) were clinically evaluated and blood samples were collected from individuals who consented to the study. Blood samples from hundred ethnically-matched unrelated healthy individuals were also collected to be used as a control for allele frequency calculation and validation of disease-associated mutation. Genomic DNA from peripheral blood samples was extracted by standard phenol–chloroform DNA extraction procedure [22].

### Genotyping

Genomic DNA from six family members was genotyped by using *FGFR3* linked microsatellite markers; D4S412, D4S2366, D4S394, D4S403, D4S419, D4S391, D4S405, and D4S1627. Standard PCR protocol was followed to amplify microsatellite markers using genomic DNA. Each reaction was carried out in 10 μl volume containing 1.5 mM MgCl_2_, 0.6 μM of each primer, 0.2 mM each dNTPs, 1 U Taq DNA polymerase and 1× PCR buffer (Bio-line, London, UK). Thermocycler conditions included an initial denaturation at 94 °C for 5 min, followed by 35 cycles of denaturation at 94 °C for 45 s, annealing at 55 °C for 45 s, extension at 72 °C for 45 s and a final extension at 72 °C for 10 min. The PCR products were separated on an 8% non-denaturing polyacrylamide gel stained with ethidium bromide and alleles were assigned through visual inspection.

### Sanger sequencing and RFLP

Intronic primers (see Additional file 1) were used to amplify all coding exons and adjacent splice sites to identify any potential pathogenic variant of *FGFR3*. Amplified PCR products were purified through QIAquick PCR Purification Kit (Qiagen, U.K.) and subjected to bidirectional Sanger sequencing using Big Dye® Terminator v3.1 cycle sequencing kit in an ABI 3130 genetic analyzer (Applied Biosystems, Foster City, CA, U.S.A).

For RFLP analysis, the candidate region of *FGFR3* was PCR amplified using genomic DNA of all available samples. Amplified products were purified and digested with site specific restriction enzyme at 37 °C for 16 h by following manufacturer’s instructions (Thermo Scientific). The digested products were separated on 3% agarose gel and genotypes were called by visual inspection under UV. To ensure the mutation does not exist in our population as a common polymorphism, hundred ethnically-matched controls were also screened.

### In silico analysis

Secondary and tertiary structure features of the wild-type/mutant proteins were predicted by Psipred [23] and I-Tasser [24, 25] respectively. RAMPAGE server [26] confirmed the accuracy of models. Pockets on three-dimensional structures of proteins were identified using CASTp server [27, 28]. Meta SNP [29], I-Mutant2.0 [30] and PredictSNP [31] were utilized to estimate the effect of mutation on protein stability and to determine whether the identified mutation has an impact on normal function of protein or not.

### Sequence acquisition

Reference sequences of *FGFR3* gene (NG_012632.1), coding nucleotides (NM_000142.4) and amino acids (NP_000133.1) were retrieved through NCBI database.

## Results

### Clinical details

**Fig. 1:**
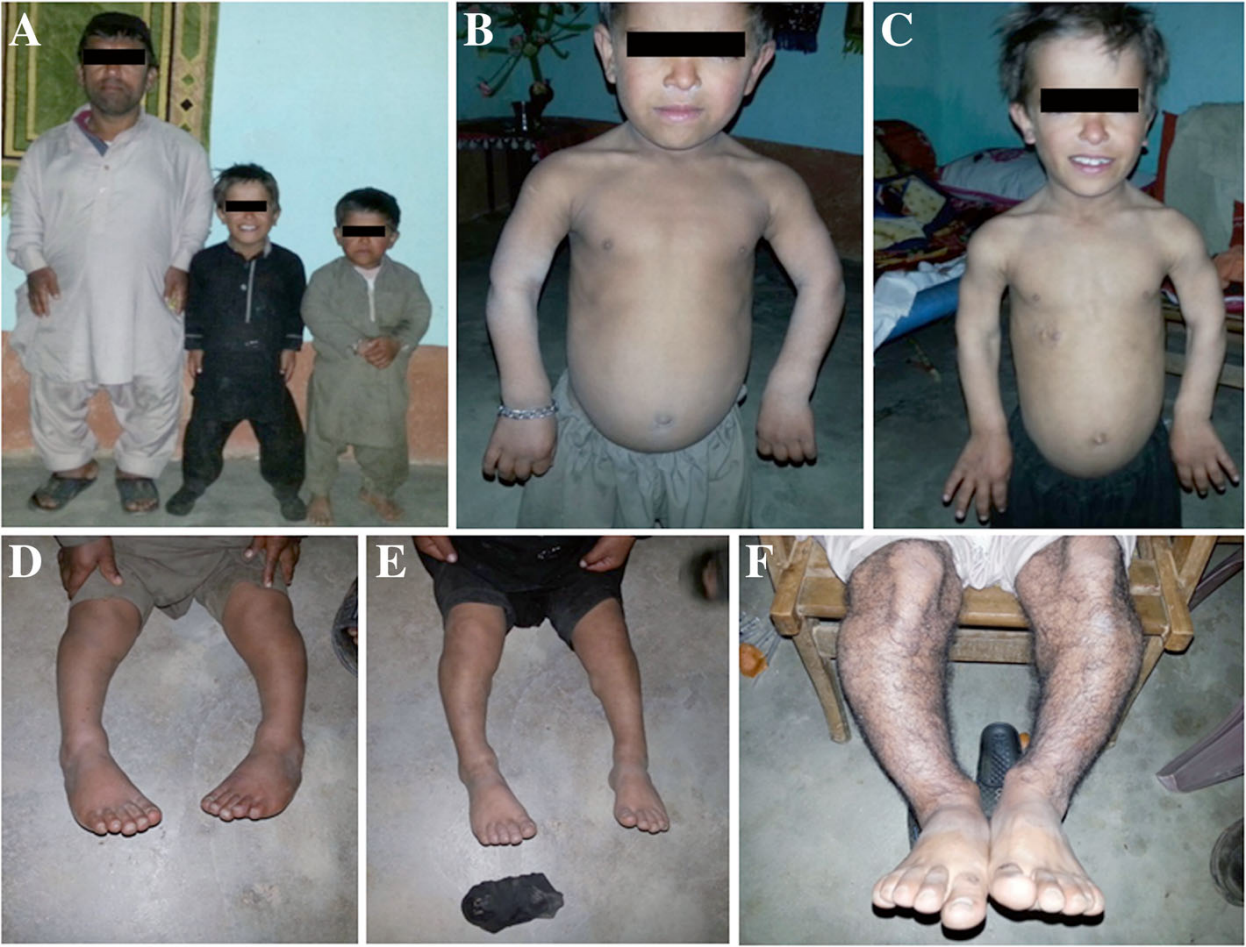
Clinical presentation of a Pakistani family with autosomal dominant achondroplasia: (**a**) Affected individuals showing short stature with near to normal head growth. Shortened hands of father (II-3) are prominent but not evident in children due to their younger ages. **b**, **c** Represent affected individuals III-1 and III-2 in the pedigree respectively; facial and skull growth pattern is near to normal with mild flat nasal bridge. Axial skeleton is less affected, whereas shortened humerus is prominent. **d**, **e** Affected individuals III-1 and III-2 in the pedigree respectively; both children are displaying bilateral bowing of legs and little feet intoeing. **f** Affected individual II-3 in the pedigree; an adult presenting characteristic bilateral bowing of tibia

Abnormal endochondral ossification of long bones was a primary prominent defect observed in all patients. This resulted in shorter and broader tubular bones, particularly the femur (thigh bone) and humerus (arm bone), compared to an average aspect ratio (length/width) of bone in a normal population (Fig. 1a-f). The growth pattern of iliac crest remained normal; however, the standing heights were dropped below the third percentile of the age.

Facial and skull growth pattern was near to normal in affected individuals. The morphology of hands was smaller than the average growth in adults only because in children it cannot be marked (Fig. 1a). The axial skeleton was relatively involved but the appendicular skeleton was more prominent to be rhizomelic (Fig. 1b and c).There was bilateral tibial bowing in all patients; however, a little element of intoeing secondary to the bowing of legs in children was clinically and physically obvious (Fig. 1d-f). The gait cycle of walk was also normal. The cognitive system of all our patients was spared. The intelligence was also within normal values; however, there was a history of gross motor developmental delay meant that children had delayed milestones during their initial development. Remaining neurological examinations were normal. The soft tissue structures of gastrointestinal system, genitourinary system, respiratory system and visual and acoustic systems all were in the normal range.

### Genotyping and mutation analysis

**Fig. 2:**
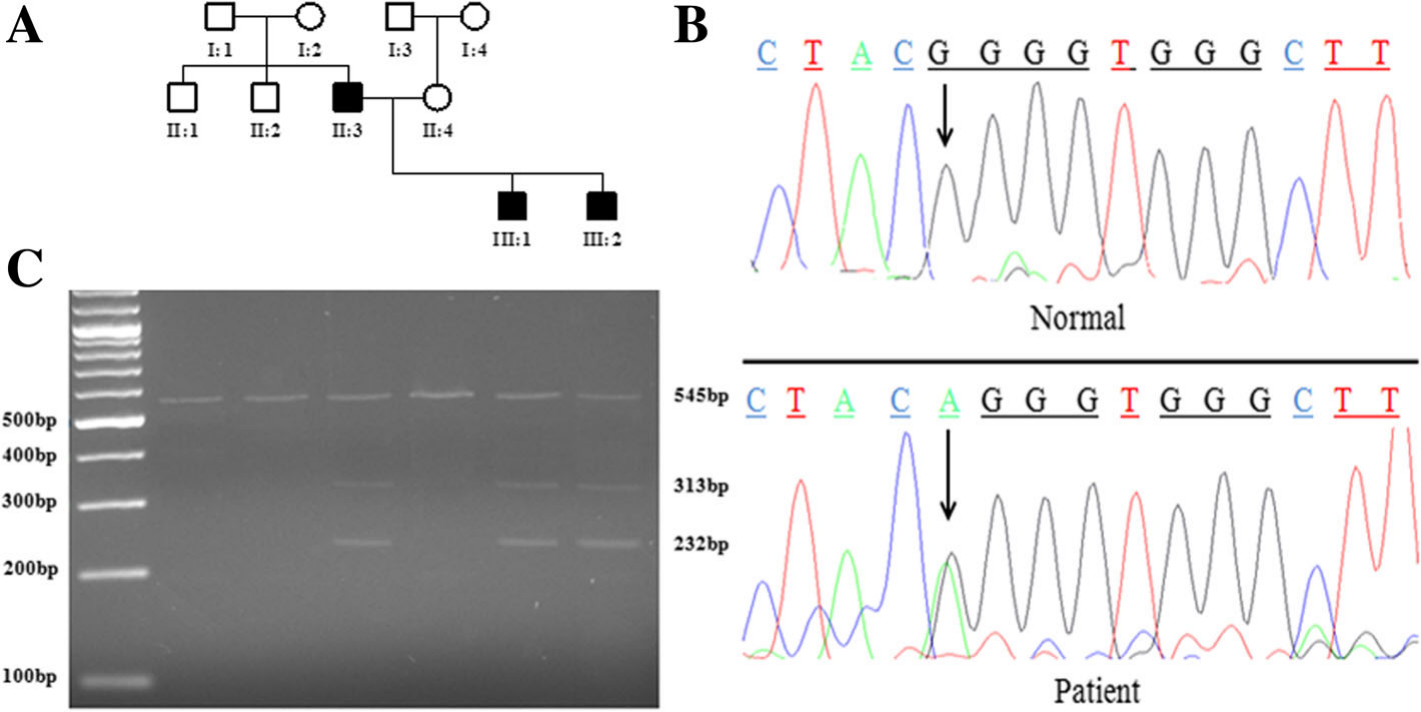
The ACH family pedigree and DNA sequence analysis: (**a**) Pedigree of a non-consanguineous Pakistani family where ACH is segregating as dominant trait. Three affected individuals (II-3, III-1, III-2) and three phenotypically healthy individuals (II-1, II-2, II-4) were analysed. **b** Sanger sequencing of *FGFR3* gene revealed a c.1138 G > A heterozygous mutation in affected individuals while homozygous wild-type allele (G/G) in phenotypically healthy individuals. Arrow indicates the site of mutation. **c** PCR-RFLP analysis; an electrophoretic banding pattern of *SfcI* digestion revealed homozygous status for all healthy individuals while heterozygous condition observed in affected individuals

Genotyping of each three affected and normal individuals (Fig. 2a) by using microsatellite markers mapped the disease at cytogenetic locus 4p16.3. The markers were fully informative and their heterozygous status in all affected individuals suggesting the disease linkage to the locus carrying previously reported *FGFR3* gene.

Sanger sequencing detected a heterozygous G > A substitution at nucleotide position 1138 (c.1138 G > A) in coding exon 8 of the *FGFR3* causing glycine replacement with arginine (p.G380R). Affected individuals were found heterozygous for the change while phenotypically healthy individuals identified with normal genotype (Fig. 2b). Identified variation was not found in a panel of hundred ethnically-matched control samples. Sequences were compared with the NCBI reference sequence; NG_012632.1.

RFLP results were analyzed by simply counting and comparing the bands sizes in healthy as well as affected individuals. Digestion with *SfcI* restriction enzyme produced expected banding pattern; a single band of 545 bp was observed in healthy individuals while three fragments of sizes 545 bp, 313 bp and 232 bp were identified in affected s individuals. The banding pattern observed in all affected individuals not only indicates the heterozygous status of the disease allele but also confirms the presence of *SfcI* restriction site created by c.1138 G > A mutation (Fig. 2c).

### In silico analysis of secondary structure features and protein stability

**Fig. 3:**
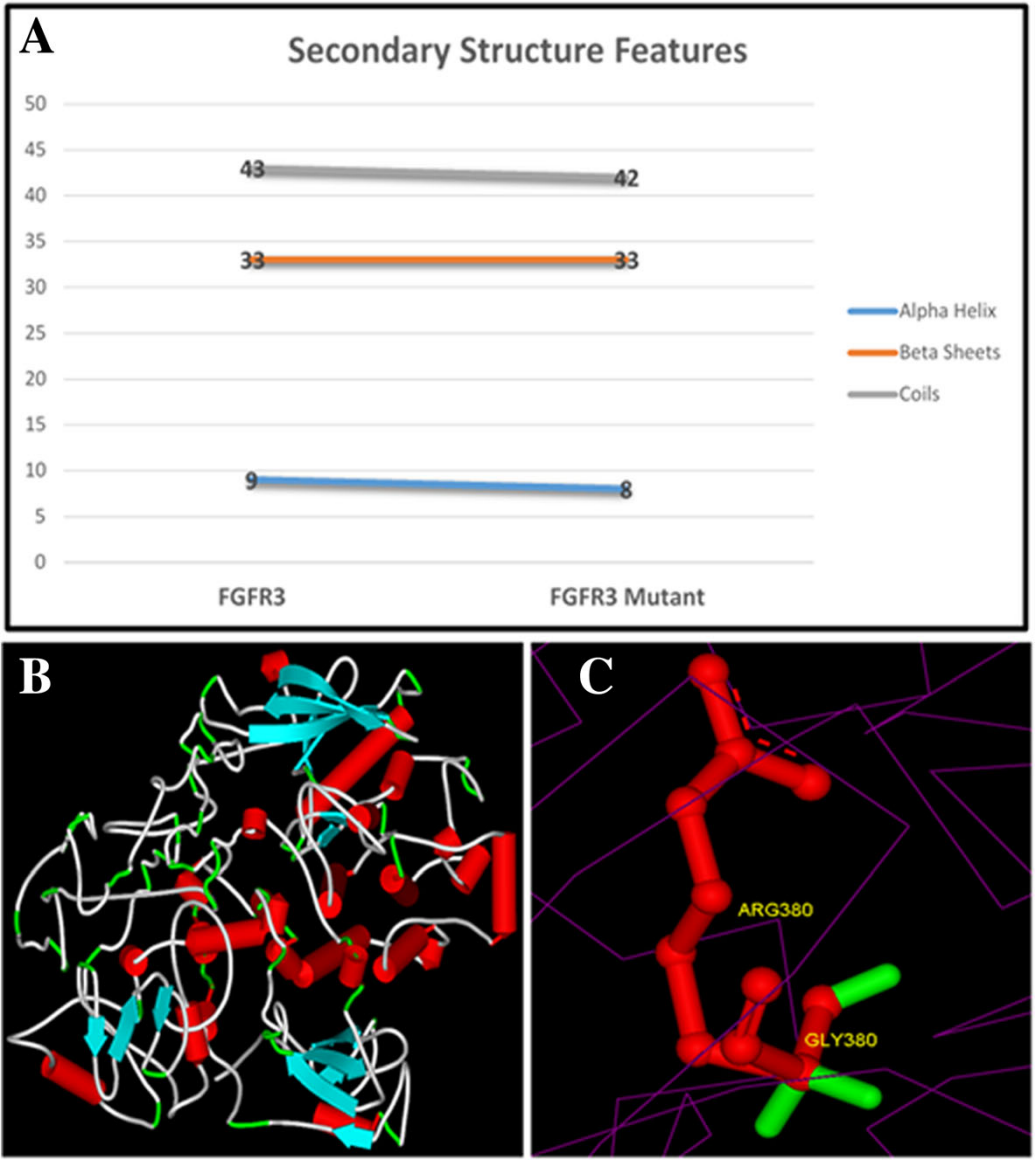
Structure modeling: (**a**) PsiPred results showing number of secondary structure features in the wild-type and mutant forms of FGFR3. **b** Three-dimensional structure prediction of wild-type FGFR3 using I-Tasser. Red cylinders showing α-helix, flattened cyan color arrows showing β-Sheets and rest tube like features are coils. **c** Superimposed three-dimensional structures of wild-type and mutant highlighting the substituted amino acid. Superimposed structures represented by purple wire display style while red ball & stick model is representing arginine which got substituted by glycine (green color stick display style) at 380 amino acid position (p.G380R)

Protein secondary structure analysis showed number of features such as alpha helices, beta sheets and coils in both forms of the protein (Fig. 3a). This shows a little variation in the number of structure features but still has a significant impact on overall conformation of the structure, hence its interaction and function. This can further be seen in three-dimensional structure modeling where normal structure has been shown in part B while mutation is highlighted in part C. Structure evaluation also indicated reliability of the predicted models by showing maximum number of residues in favored region.

**Fig. 4:**
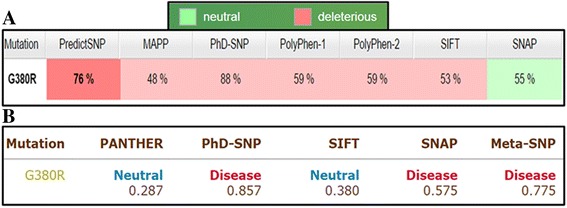
FGFR3 Mutation (p.G380R) effect prediction: (**a**) PredictSNP results. **b** Meta SNP results

As per I-Mutant2.0 calculations, protein stability decreases due to amino acid substitution. For this, two additional tools i.e. PredictSNP & Meta SNP were also utilized to analyze the effect of mutation on protein (Fig. 4a and b). PredictSNP utilizes data from different powerful predicting methods, integrates and computes the data to produce more accurate and significantly improved predictions on functional status of a protein. PredictSNP sorts out SNPs with damaging consequences as natural variant of disease phenotype. Generally, in such conditions, stability of protein is compromised. Contrary to this, SNPs are mostly classified as neutral/ benign those with no known disease symptoms. Meta SNP also hires with it different predictors (PATHER, PhD-SNP, SIFT and SNAP) to calculate mutation impact on normal protein. The values reported for each prediction are summarized in Fig. 4b. Maximum number of predictors anticipated p.G380R mutation as a disease causing which further validated our results. Reference values for prediction tools are given in Additional file 2.

## Discussion

Achondroplasia, primarily a failure of endochondral ossification in the growth plate of cartilage, defined to be associated with pathogenic mutations in the transmembrane segment of *FGFR3* gene [32, 33]. To date, eight different pathogenic mutations of *FGFR3* transmembrane segment have been shown implicated in cancer and growth disorders including ACH; however, exact pathogenetic mechanism of the disease is still unclear. In the present study, we screened a non-consanguineous Pakistani family with ACH and identified a highly recurrent heterozygous c.1138 G > A mutation of *FGFR3*. The single nucleotide substitution not only replaces glycine with arginine at codon position 380 (p.G380R) but also generates a unique restriction site for *SfcI* endonuclease. An electrophoretic banding pattern of *SfcI* digestion supports the dominant mode of disease segregation in the family. The presence of heterozygous c.1138 G > A mutation in all affected individuals, but none of the phenotypically normal members of the family along with hundred ethnically-matched healthy controls confirms the previously reported association of mutation with ACH in this Pakistani family.

The identified genetic variation was further tested through in silico analysis to calculate its impact on protein structure, stability and function attributed to disease under study. Comparative in silico analysis of varied features of wild-type and mutant FGFR3 proteins predicted significant conformation changes in the secondary protein structure. As a consequence, decreased protein stability with altered interaction and function can be expected. The Identified mutation has previously been described to be implicated in persistent ligand-independent activation of the FGFR3 protein, a deviation from its normal function as a negative bone growth regulator [4, 18]. In the present study, it was calculated through bioinformatics that the identified mutation decreases the protein stability, hence disrupted the normal functioning of FGFR3 protein completely or partially and categorized as deleterious ACH mutation.

Under normal circumstances, FGF/FGFR3 signaling initiates lateral dimerization of FGFR3 monomers to take on autophosphorylation of tyrosine residues in the intracellular domain of FGFR3 [34]. The transmembrane domain plays a critical role in this dimerization and/or activation of receptor tyrosine kinases by controlling orientation of the intracellular kinase domain [35, 36]. Once activated, FGFR3 phosphorylate cytoplasmic target proteins, leading to the activation of intracellular downstream signaling cascade. Signaling Pathways such as mitogen-activated protein kinase (Ras/MAPK), phosphoinositide 3-kinase/Akt, phospholipase C and protein kinase C pathways inhibit chondrocyte proliferation through STAT1 signaling by inducing the expression of cell cycle suppressor genes such as CDK inhibitor p21, hence negatively regulate bone growth [10, 37].

In contrast, both the deficiency of *FGFR3* or presence of gain-of-function mutations in *FGFR3* are reportedly implicated in prolonged bone growth or short stature respectively, suggesting the role of FGFR3 as limiting factor rather than a promoting agent [10, 38, 39]. Among the known mutations of *FGFR3*, c.1138 G > A substitution (p.G380R) is the most recurrent point mutation ever known in human genome accounts for almost 98% ACH cases [18, 40–42]. It is considered that p.G380R change causes hydrogen bonding between two arginine side chains that stabilizes FGFR3 dimerization in the cell membrane, leading to a constitutive ligand-independent activation of the FGFR3 [43]. Thus, uncontrollable signal transduction promotes the inhibition of cartilage growth and development by aberrantly inducing the expression of cell cycle suppressor genes [35, 43]. Many other studies have hypothesized that point mutations in the FGFR3 transmembrane regions can induce conformational rearrangements of the dimer that can affect its association strength in different states and hence represent a general mechanism of activation of such receptors [40, 43]. However, the exact mechanism of FGFR3-mediated signal transduction in health and disease is still unknown.

## Conclusion

We report a common FGFR3 substitution mutation p.G380R in three patients from a non-consanguineous Pakistani family. In view of previous reports we assume that the identified mutation might have altered the dimerization efficiency of transmembrane region leading to uncontrolled signal transduction and expression of cell cycle suppressor genes. This might clarify the decreased cell proliferation in the growth plate and would justify the basis for reduced endochondral bone growth and disproportionate short stature in ACH. Further detailed genotype-phenotype studies are expected to provide a better understanding of the role of FGFR3 in skeletal development. Our findings agree with the world-wide sharing of p.G380R mutation among achondroplasia subjects and extend the body of evidence that supports the role of *FGFR3* gene in ACH. Moreover, like many other countries, *Sfc1* restriction site in *FGFR3* can be used as a molecular diagnosis marker for ACH in Pakistan.

## Additional files

Additional file 1: Table S1. Intronic primers used to amplify coding exons of FGFR3 gene (Doxc). (DOCX 15 kb)

Additional file 2: Table S2. Reference values for Meta SNP prediction tools. (DOCX 13 kb)

## Abbreviations

ACH: Achondroplasia
CDK: Cyclin-dependent kinase
FGFR3: Fibroblast growth factor receptor 3
HCH: Hypochondroplasia
KPK: Khyber Pakhtunkhwa
MAPK: Mitogen-activated protein kinase
RFLP: Restriction fragment length polymorphism
SADDAN: Severe achondroplasia, with developmental delay and acanthosis nigricans
STAT1: Signal transducer and activator of transcription 1
TD: Thanatophoric dysplasia
