# Supplementary material for: Identification and in silico characterization of p.G380R substitution in FGFR3, associated with achondroplasia in a non-consanguineous Pakistani family
Source: Diagn Pathol. 2017 Jul 5;12:47. doi: 10.1186/s13000-017-0642-3 (PMC5499044; doi:10.1186/s13000-017-0642-3)
Supplement: Supplementary file 1 — Intronic primers used to amplify coding exons of FGFR3 gene (Doxc). (DOCX 15 kb) [file 13000_2017_642_MOESM1_ESM.docx]

# Additional file 1

**Table 1:** Intronic primers used to amplify coding exons of *FGFR3* gene.

| **Gene** | **Primer ID** | **Primer Sequence (5’-3’)** | **Ta °C** | **Product (bp)** |
| --- | --- | --- | --- | --- |
| FGFR3 | FGFR3_E1_F | CTGCCTTCCTCCTCCTGTAG | 57 | 336 |
|  | FGFR3_E1_R | CGTCACTCACACCCGGC |  |  |
|  | FGFR3-E2_F | CTGTAAACGGTGCCGGG | 58 | 398 |
|  | FGFR3-E2_R | GACCCACGCAGGGACTC |  |  |
|  | FGFR3_E3_F | GGACCCTGCCCCATCTG | 58 | 239 |
|  | FGFR3_E3_R | CCTTAGTCCCTCAGCTGCC |  |  |
|  | FGFR3_E4/5_F | AGAGGGGCCTCTGCTCC | 60 | 514 |
|  | FGFR3_E4/5_R | AGATGACGCTCAGGGGC |  |  |
|  | FGFR3_E6_F | TGGACGTGCTGGGTGAG | 60 | 397 |
|  | FGFR3_E6_R | CAACCCCTAGACCCAAATCC |  |  |
|  | FGFR3_E7_F | CCAGCCTCGATCTGTACCTT | 56 | 298 |
|  | FGFR3_E7_R | CTTGGAGCTGGAGCTCTTGT |  |  |
|  | FGFR3_E8_F | CTGTGGCTCTGGTGTCTCC | 62 | 545 |
|  | FGFR3_E8_R | AGAGAGGGCTCACACAGCC |  |  |
|  | FGFR3_E9_F | CTGTACCTCCACGCCCTG | 60 | 275 |
|  | FGFR3_E9_R | CTGACTGGTGGCTGTTTCAC |  |  |
|  | FGFR3_E10/11_F | GTGGTGGGCTGAGAGTGG | 58 | 450 |
|  | FGFR3_E10/11_R | GGACACGGGCTCCTCAG |  |  |
|  | FGFR3_E12/13_F | GGTAGGTGCGGTAGCGG | 58 | 558 |
|  | FGFR3_E12/13_R | CCAGGCGTCCTACTGGC |  |  |
|  | FGFR3_E14/15_F | GGGGTCATGCCAGTAGG | 57 | 583 |
|  | FGFR3_E14/15_R | TATTCGGGAACAGCCTGAAG |  |  |
|  | FGFR3_E16/17_F | CAGGCTGTTCCCGAATAAGG | 56 | 553 |
|  | FGFR3_E16/17_R | CACCAGCAGCAGGGTGG |  |  |

F= forward primer; R = reverse primer; Ta = optimal annealing temperature; bp = base pair

Highlighted Pink: Primers used to amplify exon 8 of the FGFR3.
