# Supplementary material for: Identification and in silico characterization of p.G380R substitution in FGFR3, associated with achondroplasia in a non-consanguineous Pakistani family
Source: Diagn Pathol. 2017 Jul 5;12:47. doi: 10.1186/s13000-017-0642-3 (PMC5499044; doi:10.1186/s13000-017-0642-3)
Supplement: Supplementary file 2 — Reference values for Meta SNP prediction tools. (DOCX 13 kb) [file 13000_2017_642_MOESM2_ESM.docx]

# Additional file 2

**Table 2**: Reference values for Meta SNP prediction tools

| **S.No.** | **Predictor Tools** | **Reference Values** |
| --- | --- | --- |
|  | PANTHER | Between 0 and 1. (If >0.5 mutation is predicted Disease) |
|  | PhD-SNP | Between 0 and 1. (If >0.5 mutation is predicted Disease) |
|  | SIFT | Positive Value (If >0.05 mutation is predicted Neutral) |
|  | SNAP | Output normalized between 0 and 1 (If >0.5 mutation is predicted Disease) |
|  | Meta-SNP | Between 0 and 1. (If >0.5 mutation is predicted Disease) |
